# Cholinergic Stimulation Improves Oxidative Stress and Inflammation in Experimental Myocardial Infarction

**DOI:** 10.1038/s41598-017-14021-8

**Published:** 2017-10-20

**Authors:** Otávio C. Bezerra, Cristiane Miranda França, Juraci Aparecida Rocha, Gizele A. Neves, Pamella Ramona M. Souza, Mariana Teixeira Gomes, Christiane Malfitano, Tatiane C. Alba Loleiro, Paulo Magno Dourado, Susana Llesuy, Katia de Angelis, Maria Claudia C. Irigoyen, Luis Ulloa, Fernanda M. Consolim-Colombo

**Affiliations:** 1 Universidade Nove de Julho (UNINOVE), São Paulo, SP Brazil; 2 Division of Biomaterials and Biomechanics, Department of Restorative Dentistry, School of Dentistry, Oregon Health and Science University, Portland, OR 97229 USA; 3 Hypertension Unit, Heart Institute (INCOR) School of medicine, University of São Paulo, São Paulo, SP Brazil; 4 Department of Physiology and Biophysics, Institute of Biomedical Sciences, University of São Paulo, São Paulo, Brazil; 5 Universidad de Buenos Aires, Buenos Aires, Argentina, Facultad de Farmácia y Bioquímica, Buenos Aires, Argentina; 6 Center of Immunology and Inflammation, Rutgerts - New Jersey Medical School, Newark, NJ 07101 USA

## Abstract

We previously reported that cholinergic stimulation with pyridostigmine (PY) induces anti-inflammatory cell recruitment soon after myocardial infarction (MI). In this study, we evaluated the anti-inflammatory effects of PY during the proliferative phase of cardiac repair by analyzing the infiltration of macrophages, Treg lymphocytes, oxidative stress and inflammatory cytokines. Wistar rats underwent control sham surgery or ligation of the left coronary artery and were randomly allocated to remain untreated (untreated infarcted group, I) or to receive PY (30 mg·kg(−1)·day(−1)) in the supplied water (infarcted treated group, I + PY). Blood pressure and heart rate variability were registered at day 5 post-MI. The animals were euthanized 7 days after thoracotomy, when the hearts were removed and processed for immunohistochemistry (CD68, CD206, FOXP3), cytokines (IL-1β, IL-6, IL-10, TNF-α) and oxidative stress (superoxide dismutase, catalase, glutathione peroxidase, lipidic and protein peroxidation). PY treatment increased parasympathetic modulation, M2 macrophages and the anti-oxidant enzyme activity but reduced protein oxidation (carbonyls) and the concentration of IL-1β, IL-6, TNF-α and IL-10. Cholinergic stimulation induces parasympathetic neuro-immune modulation and anti-inflammatory cell enrollment as well as prevents oxidative stress and cytokine production after MI.

## Introduction

Myocardial infarction (MI) triggers an intense aseptic inflammatory response leading to either local fibrosis with myocardial rupture or expansion of the myocardial damage with severe remodeling and heart failure^1,2^. Both processes involve the overproduction of inflammatory cytokines and the recruitment of immune cells into the ischemic area in response to the production of reactive oxygen species (ROS)^3,4^.

Inflammatory cytokines appear within the first hours after MI and they return to baseline levels after one week, but if the infarcted area is large, these cytokines persist or have a second wave of stimulation^5^.

The reactive oxygen species (ROS) released during the acute phase of the ischemic damage are well known for their critical role activating the immune system and triggering tissue repair^4^. However, high levels of ROS over long period of time causes oxidative injury as a secondary damage by reaching vital cellular constituents, such as lipids, proteins and DNA. This cellular damage exacerbates inflammation and interferes with tissue repair^3,6^. Tissue damage induced by ROS depends on the potential of the cellular antioxidant enzymes to control oxidation^7^. The imbalance between ROS levels produced during metabolism and eliminated by the antioxidant system is termed “*oxidative stress*”. This oxidative stress has major clinical implications as it contributes to the pathology of myocardial infarction as shown in both experimental^3,8^ and clinical patients after MI^9^. There is a strong association between the oxidative stress, inflammation, development of adverse cardiac remodeling^4,5^ and heart failure after MI^10^.

Therefore, novel therapeutic strategies against myocardial infarction intend to control both the inflammatory response as well as the oxidative stress. Based on this approach, one of the most important mechanisms modulating cytokine production by the immune cells is the cholinergic anti-inflammatory pathway^11^. Recent studies revealed that the vagus nerve, the main cholinergic nerve of the parasympathetic nervous system connecting the central nervous system with the viscera, controls the innate immune responses to bacterial endotoxin^11–13^. Given that the vagus nerve releases acetylcholine, this mechanism has been called the “cholinergic anti-inflammatory pathway” and it is mediated by the activation of nicotinic cholinergic receptors in the immune cells^13,14^. Despite its recent identification, growing evidence from different groups indicate that vagal nerve stimulation controls inflammation not only in infectious disorders but also in aseptic conditions such as hemorrhage and reperfusion and cerebral ischemia^15^. These results have inspired several groups to propose vagal nerve stimulation as a potential therapeutic approach to control inflammation after myocardial infarction^16,17^. Vagal nerve stimulation (VNS) limits infarct size and the inflammatory response to myocardial ischemia and reperfusion injury^18^, and modulates cardiac redox status thereby suppressing ROS generation in an experimental model of heart failure caused by MI^19^. Furthermore, VNS exerts cardio protection via amelioration of cardiac mitochondrial dysfunction in an acute ischemic-reperfusion model^20^. However, vagal nerve stimulation has limited clinical implications because it requires surgical isolation and electrical stimulation of the vagus nerve.

We recently reported that pharmacological stimulation of the vagus nerve can provide clinical advantages for treating myocardial infarction^21,22^. Pyridostigmine bromide (PY) is a well-characterized potent and selective peripheral anticholinesterase inhibitor. PY enhances cholinergic modulation of the immune cells by preventing vagal-derived acetylcholine degradation. Given that pyridostigmine bromide is clinically used and proven safe, we reasoned that it may represent a pharmacological strategy to mimic vagus nerve stimulation and control inflammation in myocardial infarction. We and other groups showed that treatment with pyridostigmine (PY) prevents ventricular dysfunction during the onset of heart failure after myocardial infarction in different experimental models^22–24^. Most recently, we also reported that PY administration also induces specific cellular responses by enhancing the recruitment of anti-inflammatory cells^25^. The PY treated infarcted rats showed a significant increase in M2 macrophages and FOXP3^+^ cells in the infarcted and peri-infarcted areas. There is even a systemic effect in circulating immune cells and PY increased the proportion of circulating Treg (CD4^+^CD25^+^FOXP3^+^) cells, and decreased the proportion of conventional T (D25^+^FOXP3^-^) cells as observed three days after the ischemia^25^. Despite their implications, little is unknown about how these cellular responses can affect the oxidative and immune responses about a week after the ischemic event during the proliferative phase of cardiac tissue repair.

Considering these data, we hypothesized that PY increases the number of anti-inflammatory immune cells, reduces oxidative stress and cytokine production in the myocardium at the proliferative phase of cardiac repair after a permanent ischemic injury. Hence, we counted the immune cells (total and M2 macrophages and Treg lymphocytes), quantified pro- and anti-inflammatory cytokines (IL 1-β, IL-6, TNF-α, IL-10), measured parameters of cellular antioxidant enzymes superoxide dismutase (SOD), catalase (CAT) and glutathione peroxidase (GPx), and determined the levels of lipid peroxidation (TBARS) and protein oxidation (carbonyls) of the left ventricle of infarcted rats treated with PY at day 7 post-MI. Moreover, structural and functional cardiac parameters were obtained with echocardiography.

## Results

The average body weight was not different among the experimental groups (S = 239 ± 20 g; I = 242 ± 26 g; I + PY = 214 ± 36 g). Water consumption was similar among all the groups (52 ± 10 mL/d, 48 ± 7 mL/d; 47 ± 8 mL/d, respectively). Treated animals had a PY intake of 8.4 ± 2.8 mg/day (corresponding to approximately 30.7 mg/kg/day).

### Hemodynamic and autonomic measurements

Awake hemodynamic and autonomic measurements, performed on day 6 following thoracotomy, are presented in Table 1.

**Table 1:** Hemodynamic and cardiovascular autonomic values in studied groups.

	S	I	I + PY
**Hemodynamic**			
SBP (mmHg)	121 ± 2.3	117 ± 1.6*	114 ± 3.8
DBP (mmHg)	82 ± 0.9	88 ± 0.3*	83 ± 1.2^#^
HR (bpm)	337 ± 11.9	382 ± 13.4*	372 ± 8.4*
**HRV variability (**frequency-domain**)**			
LF (abs)	1.5 ± 1.7	5.5 ± 4.3	1.4 ± 1.4
HF (abs)	6.4 ± 3.7	13.7 ± 8.7	7.0 ± 1.9
LF (NU)	16.8 ± 3	28.7 ± 5*	14.7 ± 1^#^
HF (NU)	83.2 ± 3	71.3 ± 5*	85.3 ± 1^#^
LF/HF	0.2 ± 0.04	0.4 ± 0.1	0.2 ± 0.01

Abbreviations: SBP = systolic blood pressure, DBP = diastolic blood pressure, HR = heart rate, LF = low-frequency band (0.20–0.75 Hz), HF = high-frequency band (0.75–4.0 Hz). Values are expressed as mean ± SEM.

*P < 0.05 vs Sham group; ^#^P < 0.05 vs MI (n = 5, for each group).

Systolic blood pressure was significantly decreased but the heart rate was significantly increased in both the I and I + PY groups as compared to the sham group. Diastolic blood pressure was significantly higher in the I group as compared to the sham group. Pyridostigmine was very efficient preventing this effect, and diastolic blood pressure in the I + PY group was lower than in the I group but statistically similar to that in the sham group. This data indicated that pyridostigmine prevented the MI-induced increase in diastolic blood pressure.

Frequency domain analyses of heart rate variability are also shown in Table 1. These results show that myocardial infarction in the I group increased normalized LF-band values (which represents parasympathetic modulation) and lowered normalized HF-band values (which represents sympathetic modulation) leading to a higher LF/HF ratio representing a sympatho-vagal balance in the I group as compared to sham group. PY significantly increased the HF-normalized components and decreased the LF component of HRV in the I + PY group when compared to the I group. Furthermore, sympatho-vagal balance, expressed as the LF/HF ratio, was significantly reduced when compared to the I group. Therefore, the administration of PY to the infarcted rats maintained the sympato-vagal balance close to the levels observed in the sham animals.

### Echocardiographic Evaluation

Anesthetized rats were submitted to an echocardiographic evaluation six days after MI or sham surgeries, and the measurements are presented in Table 2.

**Table 2:** Echocardiographic parameters in studied groups.

Eco parameters	S	I	I + PY
MI area (%)	0	48.6 ± 14.3*	29.6 ± 2.14*
LV EF (%)	43.1 ± 2.5	25.4 ± 1.7*	24.6 ± 1.8*
LV FAC (%)	61.0 ± 6.1	28.7 ± 3.6*	36.4 ± 2.2*
LV mass (g/Kg)	3.6 ± 0.24	4.7 ± 0.5	3.7 ± 0.21
LAD (cm)	0.43 ± 0.01	0.48 ± 0.04	0.44 ± 0.02
LVDD (cm)	0.78 ± 0.04	0.88 ± 0.09	0.80 ± 0.03
LVSD (cm)	0.44 ± 0.02	0.66 ± 0.07*	0.60 ± 0.02*
LVD area (cm^2^)	0.48 ± 0.06	0.64 ± 0.08	0.60 ± 0.04
LVS area (cm^2^)	0.19 ± 0.05	0.46 ± 0.06*	0.38 ± 0.02*
ST vol (ml)	0.19 ± 0.02	0.13 ± 0.04*	0.19 ± 0.0*^#^
E/A	2.2 ± 0.31	4.0 ± 0.25*	2.6 ± 0.30^#^
IVRT/RR	0.09 ± 0.02	0.08 ± 0.01	0.09 ± 0.01

MI = myocardial.

LVEF = left ventricular ejection fraction.

FAC = fractional area change.

LVFAC = left ventricular.

LAD = left atrial diameter.

LVDD = end-diameter during diastole.

LVSD = left ventricular end systolic diameter.

LVD area = left ventricular diastolic area.

LVS area = left ventricular systolic area.

E/A = E wave A wave ratio.

IVRT/RR = isovolumetric relaxation and time RR interval ratio.

Values are expressed as as mean ± SEM.

*P < 0.05vs Sham group; ^#^p < 0.05 vs I group; (n = 5, for each group).

Compared to the S Group, the infarcted groups (I and IP) showed a similar area of hypokinesia/akinesia. Furthermore, left ventricular systolic diameter and area were significantly increased in both infarcted groups compared to S group (Table 2). As a consequence, infarcted groups had a significant reduction of LV systolic function, assessed by left ventricular ejection fraction (LVEF) and fractional area change (FAC). Infarcted rats treated with PY, compared to untreated infarcted animals, had a significant increase in left ventricular stroke volume, associated with tendency to a higher FAC. The diastolic function evaluation demonstrated that I group compared to the S group had a significant higher E/A ratio (early filling wave/late atrial contraction wave). However, the infarcted treated group (IP) compared to non-treated rats (I group) showed a significant decrease of the E/A ratio. The left ventricular isovolumetric relaxation (IVTR) was not different among groups (Table 2).

### Infiltration of macrophages and Treg cells in the infarcted and peri-infarcted zones

**Figure 1:**
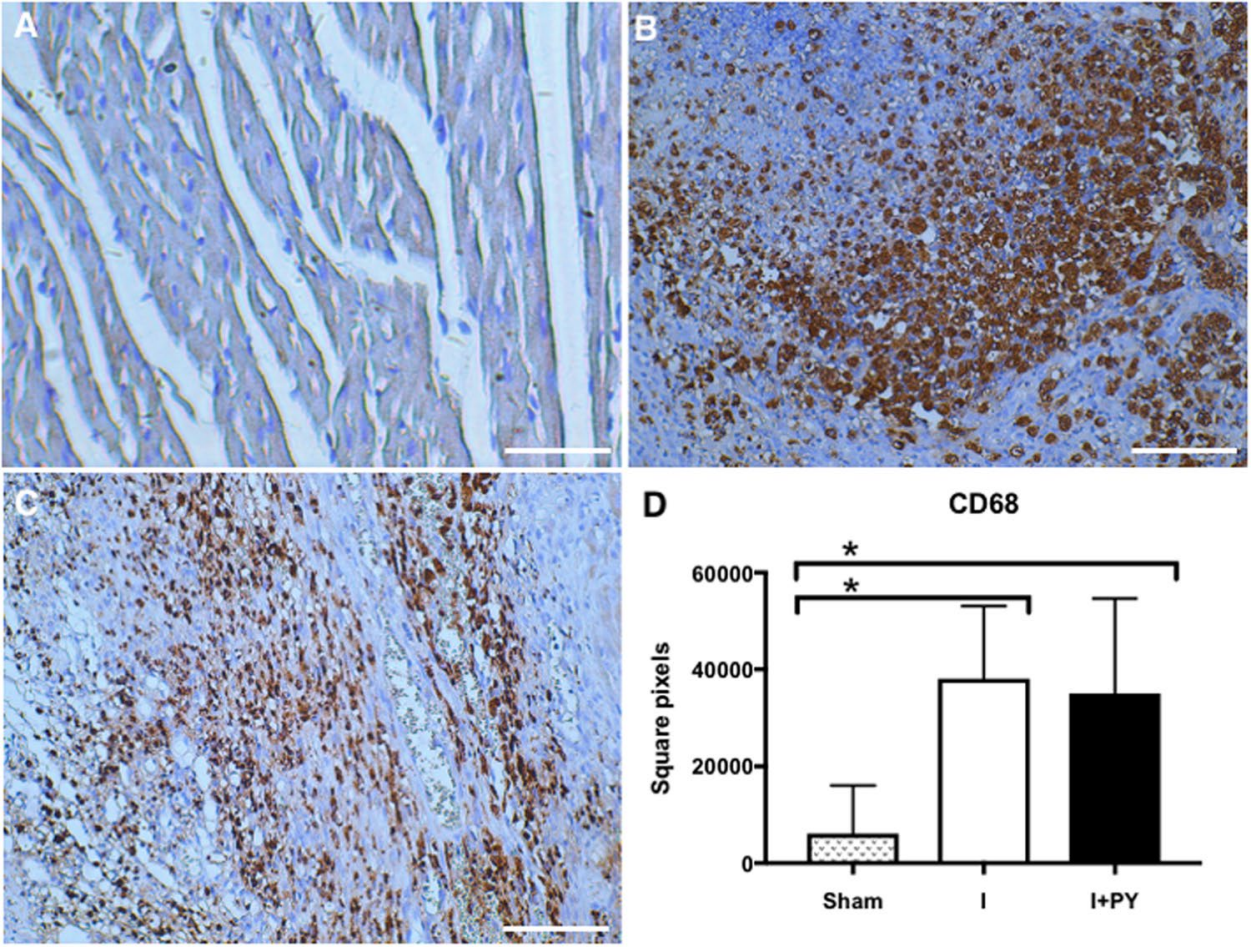
CD68 + cell count (Total macrophages). The macrophages were stained in brown, and the total area occupied by these cells was measured. Both I and I + PY groups showed dense infiltration of CD68 + cell within the infarcted and peri-infarcted zone with no statistical difference (*P* = 0.9, Kruskal-Wallis test). As expected, both groups were statistically different from the Sham group (*P* < 0.05, Kruskal-Wallis test completed by Dunn’s multiple comparison test). Values are expressed as mean ± SEM. (Immunohistochemistry, DAB, scale bar 400 μm).

**Figure 2:**
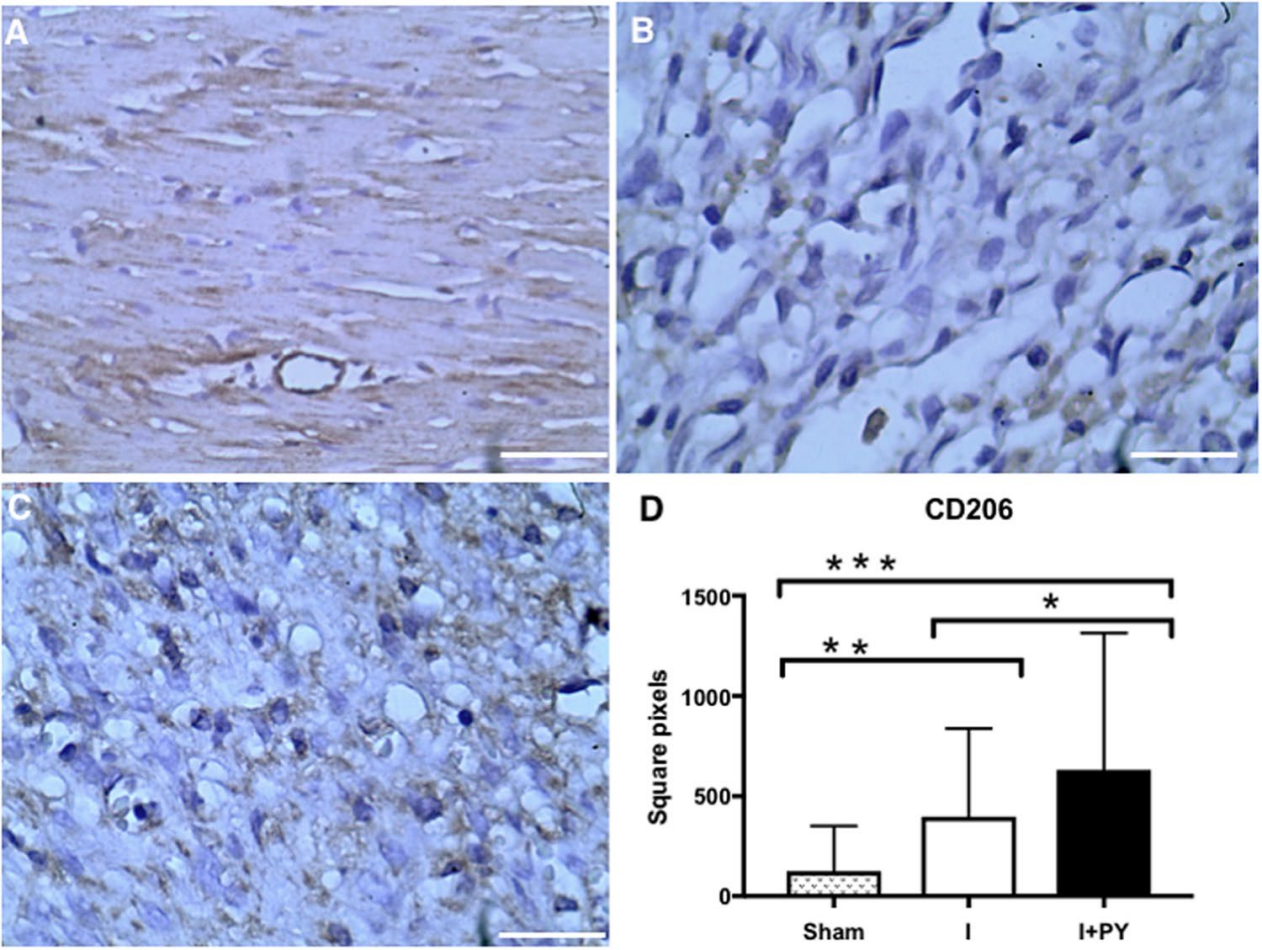
M2 macrophage (CD206+) cell count. The M2 macrophages were stained in brown, and the total area occupied by these cells was measured. Sham animals presented no M2 macrophages; I + PY group showed higher infiltration of CD206 + cell within the infarcted and peri-infarcted zone when compared to the I group (*P* = 0.04, Kruskal-Wallis test). The I group and I + PY were statistically different from the Sham group (*P* = 0.003 and *P* < 0.0001, respectively, Kruskal-Wallis). Values are expressed as mean ± SEM. (Immunohistochemistry, DAB, scale bar 400 μm).

The size of the infarcted area was analyzed both macro and microscopically. There were no significant differences between the infarcted groups (36 + 6 vs 32 + 3%, for I and I + PY groups respectively). The sham group presented few resident macrophages mainly next to blood vessels. There was a vast infiltration of macrophages into the infarcted and peri-infarcted zones at day 7 post-MI in both I and I + PY groups. The absolute count of CD68 + macrophages was statistically similar in I and I + PY groups (*P* = 0.9). Of note, the CD68 + cells in the I group were diffusely localized within the tissue, while those in the I + PY group were mainly concentrated in the peri-infarcted area (Fig. 1). On the other hand, CD206^+^ M2-macrophage counts were significantly higher in the infarcted and peri-infarcted zones of the I + PY group when compared to those in the I group (*P* = 0.04; Fig. 2).

**Figure 3:**
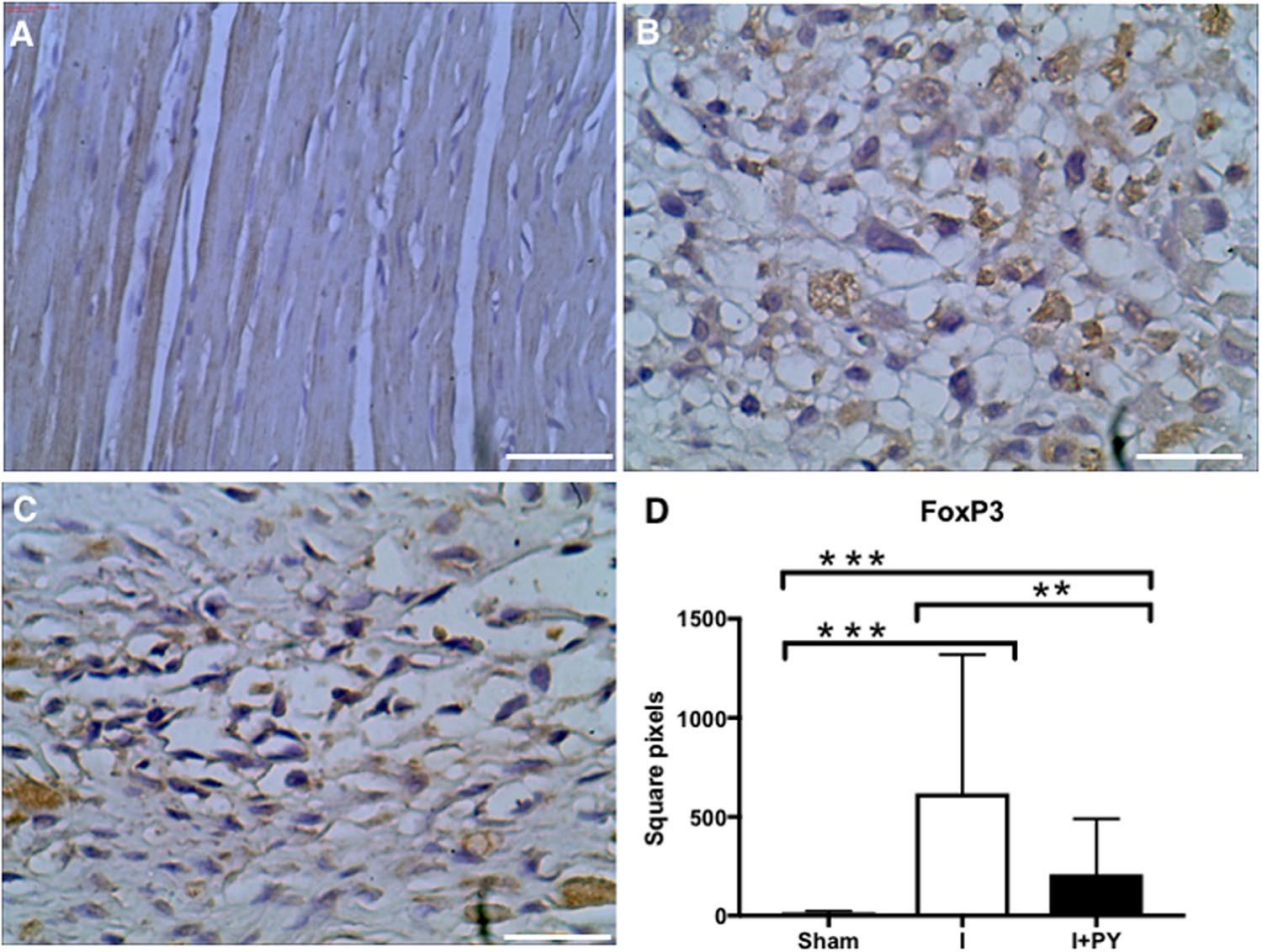
Treg cells (FOXP3^+^ lymphocytes) cell count. Treg cells were stained in brown, and the total area occupied by these cells was measured. Sham animals presented no Treg cells; I + PY group showed a higher infiltration of FOXP3^+^ cells within the infarcted and peri-infarcted zone when compared to I groups (*P* = 0.007, Kruskal-Wallis test). As expected, I group and I + PY were statistically different from the Sham group (*P* < 0.0001, Kruskal-Wallis test). Values are expressed as mean ± SEM. (Immunohistochemistry, DAB, scale bar 400 μm).

In order to study the cellular immune response, we evaluated the FOXP3-expressing T lymphocytes in the myocardium. FOXP3 is critical transcriptional factor essential for the formation of the T regulatory lymphocytes regulating inflammatory responses. As expected, the sham group did not show T lymphocytes in the heart. The I group had a higher infiltration of FOXP3 + cells within the infarcted and peri-infarcted area (*P* = 0.007). Although I + PY group had FOXP3 + cell infiltration, it was significantly lower than that in the I group (Fig. 3).

### Antioxidant enzyme activity

**Figure 4:**
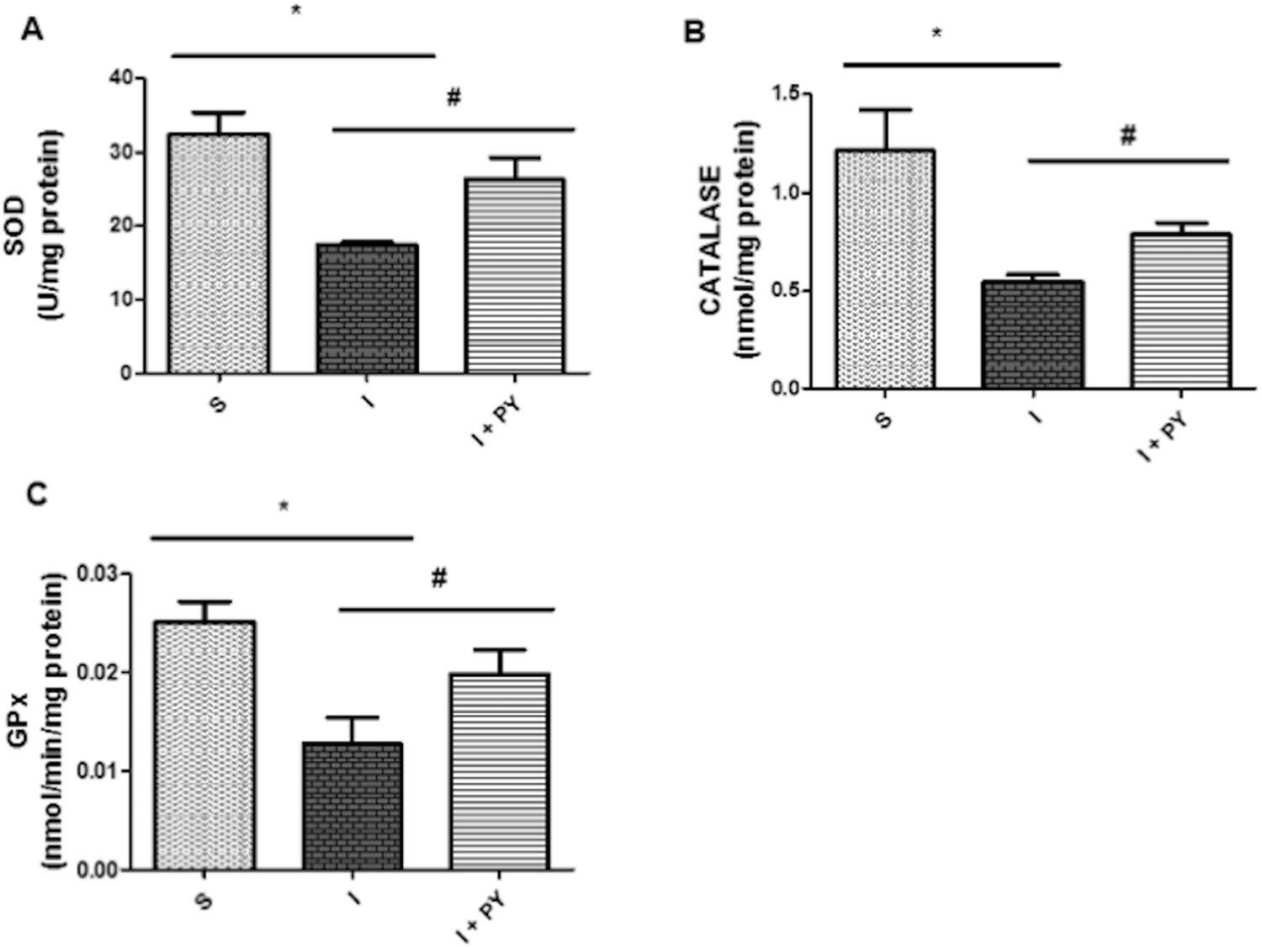
Antioxidant enzyme activity. Superoxide dismutase activity (SOD) (**A**), Catalase (CAT) (**B**), and GPX activity (**C**) were significantly lower in the infarcted rats (I group) when compared to sham animals (S group). Infarcted rats treated with PY (I + PY) presented a significantly higher activity of all anti-oxidant enzymes when compared to the untreated rats. Values are expressed as mean ± SEM. *P < 0.05 vs Sham; ^#^ *P* < 0.05 vs I (n = 7) for each group (two-way ANOVA test with Tukey as post hoc).

All the antioxidant enzymes analyzed had a similar behavior as shown in Fig. 4. Superoxide dismutase activity (SOD) activity was significantly lower in the I group. Pyridostigmine treatment significantly increased the SOD in the I + PY group when compared to the I group. Indeed, SOD values were not significantly different when I + PY and S groups were compared (Fig. 4A). Myocardial Infarction induced a significantly lower catalase (CAT) activity in the I group when compared to the sham group. CAT activity was significantly higher in the I + PY group as compared to the I group, but it was still lower than the sham group (Fig. 4B). GPX activity was significantly lower in the I group when compared to the S group. However, it was significantly higher in the I + PY group reaching values statistically similar to those observed in the sham group (Fig. 4C).

### Lipid peroxidation

**Figure 5:**
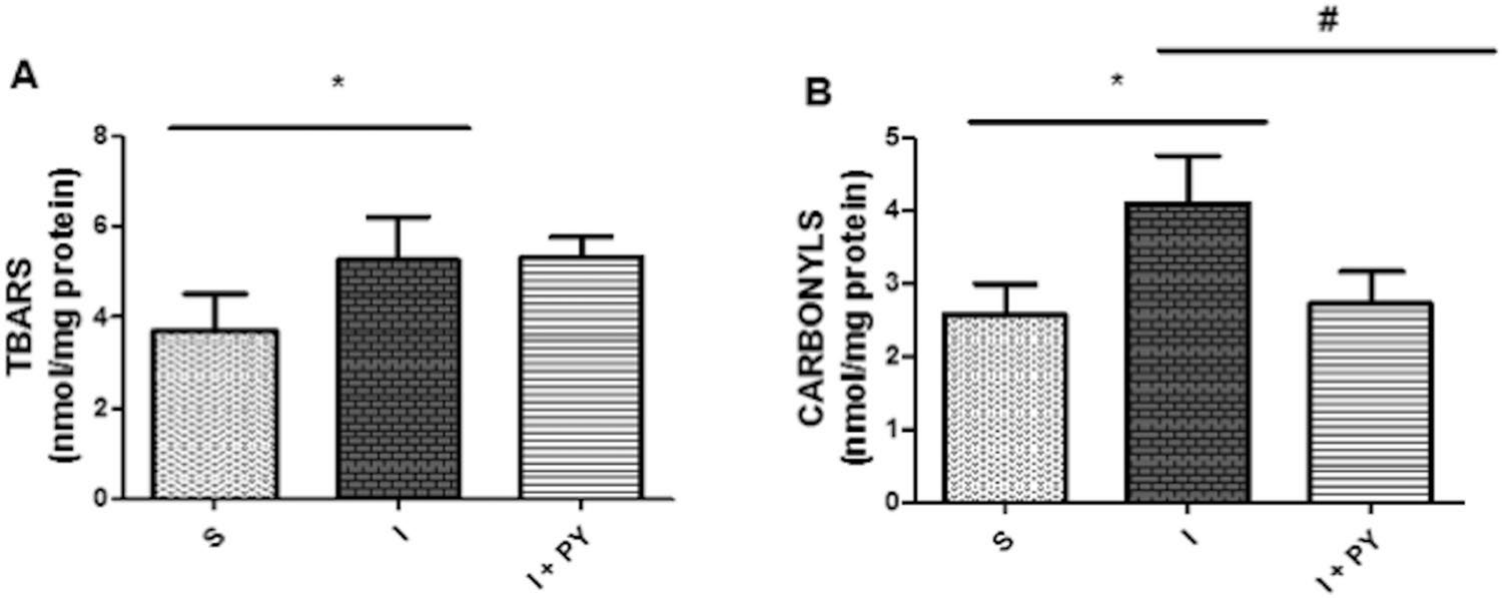
Lipid peroxidation and protein oxidation. Lipid peroxidation, obtained by measurement of TBARS, increased similarly in both infarcted groups (I and I + PY) t when compared to the S group (**A**). Protein oxidation, obtained by measurement of carbonyl groups (carbonyls) was significantly higher in the I group when compared to S group. However, in the I + PY group carbonyls were significantly lower when compared to I group, reaching values similar to those of the S group. (**B**). Values are expressed as mean ± SEM. *P < 0.05 vs Sham; ^#^ *P* < 0.05 vs I (n = 7) for each group, (two-way ANOVA test with Tukey as post hoc).

Lipid peroxidation (LPO), obtained by measurement of TBARS, increased similarly in both infarcted groups (I and I + PY) as compared to the sham group (Fig. 5A).

### Protein oxidation

Protein oxidation, analyzed by measurement of carbonyl groups (carbonyls), was significantly higher in the I group when compared to sham group. Carbonyls were significantly lower in the I + PY group the carbonyls reaching values similar to the Sham group (Fig. 5B).

### Cytokines Measurement

**Figure 6:**
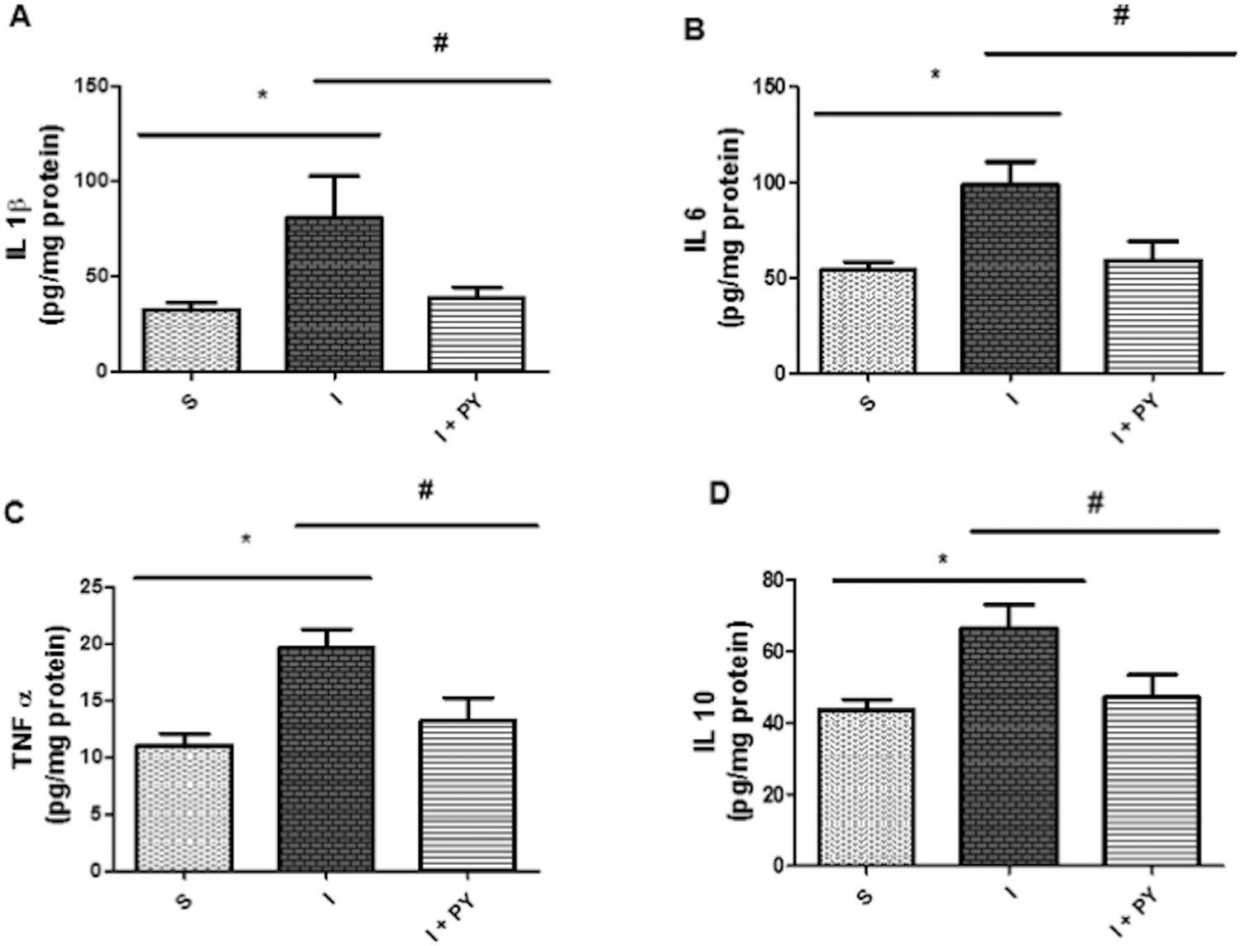
Cytokine concentrations. When compared to the S group, the I group, presented significantly higher concentrations of the pro-inflammatory cytokines IL-1β (**A**), IL-6 (**B**) and TNF-α (**C**), and anti-inflammatory cytokine IL-10 (**D**). Conversely, I + PY animals presented significantly lower values when compared to I animals. Furthermore, no differences in cytokines concentrations were observed when I + PY was compared to S group. Values are expressed as mean ± SEM. *P < 0.05 vs Sham; ^#^ *P* < 0.05 vs I (n = 7) for each group (two-way ANOVA test with Tukey as post hoc).

Left ventricle cytokine concentrations are shown in Fig. 6. Myocardial infarction in the I group significantly increased the concentrations of all the inflammatory cytokines analyzed, including IL-1β (Fig. 6A), IL-6 (Fig. 6B) and TNF-*α* (Fig. 6C), as well as anti-inflammatory cytokine IL-10 (Fig. 6D). Conversely, I + PY animals presented significantly lower values of all these immune cytokines reaching values statistically similar to that in the sham group.

## Discussion

This study shows, for the first time, that cholinergic stimulation with pyridostigmine (PY) increased M2-macrophages in the injured myocardial, improved redox state, and decreased cytokine concentration at the proliferative phase of cardiac repair after myocardial infarction. The infarcted rats treated with PY have higher activity of antioxidant enzymes (SOD, catalase, GPX), lower protein oxidation (carbonyls), and lower concentration of pro- (IL-1β, IL-6 and TNF-α) and anti-inflammatory (IL-10) cytokines in the left ventricular tissue as compared to the untreated infarcted rats. The total count of macrophages in the myocardium was similar in both infarcted groups, but the number of M2-macrophages with anti-inflammatory profile was increased in the PY-treated animals when compared to the untreated infarcted rats. Besides, the amount of Treg cells (FOXP3 + lymphocytes) in the infarcted myocardium was similar in both infarcted groups regardless of the treatment with pyridostigmine. These findings correlated with higher parasympathetic and lower sympathetic modulation as inferred by heart rate variability in PY treated rats. Our data concurs with previous studies indicating that pyridostigmine can improve the outcome from myocardial infarction^21,22^. However, our present study provides critical new information showing that pyridostigmine induce both anti-oxidative and anti-inflammatory effect improving ventricular function and remodeling after myocardial infarction.

Post-myocardial wound healing consists of a number of complex inflammatory events that are both time- and cell-type-dependent^26^. The infarcted rats treated with PY have more M2-macrophages and FOXP3^+^ T lymphocytes in the MI zone at day 3 post-MI^25^. This study shows that PY induces a lasting effect, and PY-treated animals still have higher M2-macrophages but significantly lower Treg counts in the MI zone at day 7 post-MI. One attractive hypothesis is to propose that this polarization of the macrophages from M1 to M2 may be induced by the increase of Treg cells that we reported day 3 post-MI^27,28^. This Treg response and subsequent polarization of macrophages can have significant clinical implications attenuating inflammatory cytokines^29^.

We demonstrated that PY-treated infarcted animals had a significant higher activity of antioxidant enzymes (SOD, Catalase, GPX) and lower protein oxidation (carbonyls). One prior study showed that vagal nerve stimulation altered the myocardial redox status in mice with chronic heart failure following MI^19^. The suppressed myocardial ROS over-production was mediated by inhibition of sympathetic drive and by the potential of acetylcholine to inhibit the formation of free radical involving NADPH oxidase activation, NO production, and myocardial oxygen consumption^19^. Another study revealed that vagal stimulation exerts cardioprotection by preventing cardiac mitochondrial dysfunction in swine model of myocardial ischemia and reperfusion injury^20^.

Our study also shows that the I + PY group had significant lower levels of all the immune cytokines analyzed including inflammatory TNF-α, IL-1 and IL-6, and anti-inflammatory cytokine IL-10. Either acutely or chronically, the release of pro-inflammatory cytokines adversely affects LV function, exerting a negative inotropic effect^3,5,10^. High cytokines levels, such as IL-1, are associated with ventricular diastolic diameter increase and collagen deposition in the infarcted area after several weeks of MI, and as such, IL-1 induces abnormalities in cardiac metabolism and promotes myocardial remodeling leading to heart failure^5,10^. Furthermore, we may also consider the crucial role of TNF-α levels, IL-6 and other cytokines in the development of heart failure^30^. In the I + PY group, the total macrophage number (including M1) was not different from the I group, the M2 was still high and the FOXP3^+^ was lower, suggesting that the production of cytokines by these cells may be significantly altered by PY treatment.

Therefore, the mechanisms associated with a PY-mediated decrease in oxidative stress and cytokine production observed in the present study deserves further investigation. It is well known that NF-κB proteins are of central importance in inflammation, and in other processes, such as, cell growth, survival and proliferation. Thus, NF-κB is involved in many pathological conditions, including myocardial infarction and heart failure. The NF-κB pathway could be activated mostly by the stimulation of pro-inflammatory receptors, such as the TNF Receptor, the Toll-Like receptor (TLRs), and by cytokine receptors for the interleukins. Since NF-κB is important in inflammation, some enzymes that promote the production of reactive oxygen species (ROS) are also regulated as its targets, especially in cells of the immune system. In fact, NF-κB-regulated genes play a key role in regulating the amount of reactive oxygen species, which have various inhibitory or stimulatory roles in NF-κB signaling^31^. Considering these previous findings, we hypothesized that NF-κB is an important signaling mechanism during proliferative phase of cardiac tissue repair after MI.

The intracellular mechanisms of the cholinergic anti-inflammatory pathway are important factors allowing the design of novel therapeutic strategies for acute myocardial infarction. We previously reported that similar cholinergic stimulation with acetylcholine and nicotinic agonists inhibit LPS-induced activation of NF-κB in macrophages via α7nAChR^32^. Our results were confirmed by other investigators in other cell types including human monocytes U937, microvascular endothelial HuMVEC cells and peritoneal macrophages^33^. More recent studies also indicate that cholinergic stimulation with donepezil, a well characterized acetycholinesterase inhibitor, attenuates inflammation by inhibiting the nuclear translocation of NF-κB^33^. Likewise, cholinergic stimulation with vagal stimulation (VNS) attenuates endothelial impairments and reduces inflammation in thoracic aortas by inhibiting NF-κB^34^. However, recent studies indicated that VNS-induced vascular protection in experimental myocardial injury/reperfusion is only partially mediated by the inhibition of the NF-κB^35^. Indeed, both VNS and acetylcholine inhibit MAPKs and VNS prevents inflamed colonic mucosa in inflammatory bowel disease by also inhibiting MAPKs^36^. Recent studies suggest that the efficacy of the cholinergic anti-inflammatory pathway is based on its potential to regulate several critical factors including MAPKs, JAK/stat3 and miR124^14^. Future studies are needed to determine the specific effects of PY treatment on each of these different pathways

Thus, the decrease in cytokine production at day 7 post-MI may reflect the local improvements (decreased apoptosis, reduced oxidative stress, Treg activation) that occurred days before, since a previous study has demonstrated a powerful PY-mediated- anti-inflammatory modulation at day 3 post-MI. Indeed, it is also possible that lower cytokine production would in fact be a consequence of lower cytokine release, a well-recognized feedback mechanism^3^.

After MI, there is an imbalance of the autonomic nervous system, characterized by increased sympathetic activity and reduced vagal activity, which may worsen the inflammatory response after ischemic insult^22^. Studies by Tracey’s group have shown that vagus nerve stimulation attenuates systemic inflammatory response to septic and aseptic conditions^11–14^. These results support the existence of a “cholinergic anti-inflammatory pathway”. In this sense, increasing cholinergic stimulation may be an promising strategy to protect against myocardial ischemia^16^. Direct vagal stimulation, through its nicotinic action, can reduce infarct size and risk of heart failure following MI^18–20^. Moreover, PY, a potent peripheral anticholinesterase antagonist, can represent a promising pharmacological strategy against negative changes in cardiac autonomic imbalance^21^. We and others have previously demonstrated that the PY increases vagal modulation, decreases sympathetic tone, and improves baroreflex sensitivity, while attenuating cardiac remodeling after MI^21–24^. In the present study, treatment with pyridostigmine did not change awake baseline heart rate of the infarcted animals. Similar data was also evidenced in previous studies of our group with infarcted animals^21^ as well as control animals^37^. However, the dose of pyridostigmine used was able to alter the modulation of the cardiac sympato-vagal balance, evidenced with the frequency domain spectral analysis of the heart rate variability. The main action of pyridostigmine is expected to be in the periphery since it does not cross the blood-brain barrier. By modifying the rate of degradation of acetylcholine release into the neural junction it may play a role in the dynamic properties of vagus nerve activity to the heart^38^. Moreover, we have demonstrated that pyridostigmine improved baroreflex- mediated tachycardia without changing blood pressure dynamics in normal^37^ and infarcted rats^21^. Also, using pharmacological blockade of sympathetic and parasympathetic cardiac tonus we demonstrated that intrinsic heart rate was impaired in infarcted rats, but pyridostigmine treatment returned IHR values in the infarcted treated group to values similar to those in the control group^21^. Considering that vagal tonus increased in PYR treated infarcted animals and the sympathetic tonus decreased, the observed similarity of HR in both infarcted groups are probably due to changes in the pacemaker rate control, as indicated by IHR.

PY administration interferes positively with several intracellular pathways, such as lowering activation of the TGFβ1/TAK1 pathway, which is directly involved in cardiac hypertrophy and fibrosis^17^, and improving acetylcholine-induced vascular reactivity by increasing NO^39^. It is also possible to suggest a direct effect of acetylcholine on macrophages^40^. Studies on isolated macrophages have revealed that cholinergic agonists, which pharmacologically mimic the effects of acetylcholine, significantly inhibit the production of TNF-α and other inflammatory cytokines. Acetylcholine activation of α7 nicotinic receptor triggers a complex intracellular signaling pathway including the inhibition of the NF-kB, JAK/STAT3 pathway and the miR124^14,15^.

The cardiac repercussions on the 6^th^ day after myocardial infarction were evaluated using echocardiography. This assessment has shown that the infarction resulted in systolic and diastolic dysfunction, with small but significant morphometric differences in cardiac morphology. The infarcted animals treated with pyridostigmine, compared to non-treated infarcted rats, showed a higher left systolic stroke volume, and a lower E/A ratio. Moreover, there was a tendency to improve the following parameters: MI area, LV Mass, LVDD and LVD area, and LV FAC. Besides, in the present study, similar to the results found by Benavides-Vallve *et al*.^41^ we detected a negative association between decrease in infarct size and increase in FAC%, even with less gain of left ventricle mass in the animals with pyridostigmine treatment. The FAC is obtained considering the internal area of the left ventricle during systole and diastole, and therefore, is contemplated as a more appropriate function parameter in rodents^41^. The one datum that did not show an improvement in cardiac function in response to pyridostigmine treatment was the ejection fraction, which was calculated using Teicholz’s formula. This formula does not consider the entire left ventricle cavity, but only two linear segments at the papillary muscle level. Therefore, we provided parameters that are robust and evidence potential improvements in the function and in the morphometry of the left ventricle in infarcted animals treated with pyridostigmine. These observations are in agreement with previous report from our group^25^. Considering that vagal tonus increased in PY treated infarcted animals and the sympathetic tonus decreased, the observed similarity of HR in both infarcted groups are probably due to changes in the pacemaker rate control, as indicated by IHR.

## Conclusion

PY administration increases vagal modulation, augments the number of M2 macrophages, improves redox state and lowers inflammatory cytokine levels at day 7 post-MI in rats. These findings strongly support the recently proposed concept of manipulating immune cells to control the duration and extent of the inflammatory phase following MI, particularly with the stimulation of the anti-inflammatory cholinergic pathway.

## Methods

### Experimental design

All procedures and animal care were approved by the Committee on the Ethics of Animal Experimentation at Nove de Julho University, and were performed in accordance with the *Guide for the Care and Use of Laboratory Animals* published by the US National Institutes of Health. Adult male Wistar rats (2–3 months old, 200–250 g) were housed in collective plastic cages (4 animals per cage), with the temperature-controlled (23 °C) with a 12-hour dark/light cycle and rat chow *ad libitum*. Wistar rats underwent ligation of the left coronary artery and then were randomly assigned into 2 groups (n = 14, per group): Group (I): infarcted control rats treated with vehicle; and Group (I + PY): infarcted rats treated with pyridostigmine. Group (S) represents the sham operated rats (n = 12). All animals were monitored for a total of 7 days. Rats in the S and I groups had unlimited access to tap water, and those in the I + IP group had unlimited access to water containing PY bromide (0,14 mg/ml) (Sigma-Aldrich, St Louis, Missouri) as described previously^21,25^. This treatment protocol is adequate to inhibit approximately 40% of plasma acetylcholinesterase activity^37^. Water consumption in all groups was monitored during the experimental period.

### Myocardial Infarction

Rats were anaesthetized (80 mg/kg ketamine and 12 mg/kg xylazine, i.p.) and underwent induction of MI by surgical occlusion of the left coronary artery as previously described^21,25^. A left thoracotomy was performed, the third intercostal space dissected, and the heart exposed. The left coronary artery was occluded with a single nylon (6.0) suture at approximately 1 mm distal to the left atrial appendage. The chest was then sutured. The animals were maintained under ventilation until recovery. The control group underwent the same surgical procedure but MI was not induced. Infarcted rats were randomly allocated to receive or not PY. The analytical investigators were blind of the treatment.

### Arterial catheterization, Hemodynamic measurements and heart rate variability

On day 6 after thoracotomy, the rats were anesthetized (80 mg/kg ketamine and 12 mg/kg xylazine) and a catheter filled with 0.06 mL of saline solution was implanted into the femoral artery^21,25^. Then, the arterial cannula was connected to a strain gauge transducer (Blood Pressure XDCR; Kent Scientific, Torrington, Connecticut), and arterial blood pressure (AP) and pulse interval (heart rate HR) were digitally recorded over a 30-minute period in conscious animals using a data-acquisition system (WinDaq, 2 kHz; DATAQ, Springfield, Ohio)^21,25,37^. This basal acquisition was used to evaluate the variability of pulse interval (heart rate variability, HRV).

The overall heart rate variability was measured in frequency domains using spectral estimation. For this, a 20-minute time series of pulse intervals was cubic-spline interpolated (250 Hz) and decimated to be equally spaced in time. Following linear trend removal, the power spectral density was obtained with a fast Fourier transformation using Welch´s method over 16 384 points with a Hanning window and 50% overlapping. Spectral power was calculated for very low-frequency (VLF; 0.00–0.20 Hz), low-frequency (LF; 0.20–0.75 Hz), and high-frequency (HF; 0.75–4.0 Hz) bands using power-spectrum density integration within each frequency bandwidth and a customized routine (MATLAB 6.0; Mathworks, Natick, Massachusetts). The autonomic balance was evaluated as the ratio of the absolute values of LF and HF^14,24,42^.

### Echocardiographic Evaluation

Echocardiographic evaluations were performed by a blinded observer under the guidelines of the American Society of Echocardiography. Rats were anaesthetized (80 mg/kg ketamine and 12 mg/kg xylazine, i.p.), and images were obtained with a 10–14 mHz linear transducer in a SEQUOIA 512 (Acuson Corporation, Mountain View, CA, USA). This procedure was performed six days after MI or sham surgeries in order to measure MI area and ejection fraction (EF) and to calculate the following parameters: left ventricular mass (LV mass); left ventricular end-diameter during diastole (LVDD); relative wall thickness (RWT); E wave A wave ratio (E/A); isovolumetric relaxation time (IVRT); myocardial performance index (MPI), as described in detail elsewhere^25,40^.

Through midtransversal and apical transversal views, MI size was measured by bi-dimensional echocardiogram. In diastole, three measurements of endocardial perimeter (EP) and the length of the infarcted segment (ISe) were obtained for each view. The MI size for each segment (ISi) was calculated by the equation ISi (%) = ISe/EP × 100. Total infarct size of each animal was calculated as the mean of ISi (%) of the 3 segments. MI was defined as increased echogenicity and/or change in myocardial systolic movement (hypokinesia, akinesia, or dyskinesia), in accordance with Santos *et al*.^43^. Our group^44^ and others^41^ have demonstrated strong correlations between the MI area assessed by echocardiogram and *post mortem* histological analysis, showing that this is a valid method to estimate MI area in rats.

### Immunohistochemistry for immune cells

On day 7 after thoracotomy, 7 animals per group were anesthetized (80 mg/kg ketamine and 12 mg/kg xylazine, IP) and perfused with 0.9% NaCl plus 14 mmol/LKCl solution (IV – with a pressure equal to 13 cm H_2_O,) to induce diastolic arrest, followed by a perfusion of 4% buffered formalin for tissue fixation. Harvested hearts were immersed in formalin for 24 hours. Transverse slices were processed and embedded in paraffin. Serial sections of paraffin-embedded tissues (3 µm) were placed on glass slides coated with 2% 3-aminopropyltriethylsilane (Sigma-Aldrich, St. Louis, Missouri) and deparaffinized in xylene, then immersed in alcohol and incubated with 3% hydrogen peroxide diluted in Tris-buffered saline (TBS; pH 7.4). The sections were blocked by incubation with 3% normal goat serum for 20 minutes and immersed in a citrate buffer (pH 6.0; Sigma-Aldrich Co., St. Louis, Missouri) at 95 °C for 20 minutes for antigen retrieval. Nonspecific signals were blocked using specific antibody diluents (Antibody Diluent, cat S0809, Dako Corp., Glostrup, Denmark). The slides were then incubated with the following antibodies: FOXP3 (T reg cells - cat 22510, Abcam, Cambridge, Massachussets), CD68 (pan macrophage marker - cat 31630, Abcam), CD206 (M2 macrophage marker - cat 64693, Abcam).

The samples were kept overnight at 4 °C in a humidified chamber. The sections were then washed with TBS and incubated with N-Histofine Simple Stain (Nichirei Biosciences Inc., Tokyo, Japan) for 30 minutes and then incubated in 3,3′-diaminobenzidine in a chromogen solution (Dako Corp.) at room temperature for 2 to 5 minutes. The sections were then stained with Mayer’s hematoxylin (Sigma-Aldrich Co.) and covered. For the negative controls, the primary antibodies were replaced with 1% PBS/bovine serum albumin and non-immune mouse serum (X501-1, Dako Corp.)

### Cell counts in the infarcted and peri-infarcted zones

Infarction size was assessed by histology with measurements of epicardial and endocardial circumferences of the infarcted in relation to the total circumferences of the left ventricle (% infarcted area).

Ten consecutive microscope fields (magnification: 400x) of the infarcted and peri-infarcted zones were photographed (Leica Microsystems, Wetzlar, Germany). An experienced pathologist, with no prior knowledge of the samples, analyzed the images and counted the cells with the aid of the ImageJ 1.45 software (NIH, Bethesda, Maryland), using the “cell-counter” plug-in^25^. A set of 7 animals in each group was euthanized by decapitation on day 7 after thoracotomy, in order collect fresh heart for cytokine and oxidative stress analyses.

### Cytokine Measurements

Measurement of the cytokines was performed in samples of the LV protein by ELISA using Duo-set available kits for IL-1β and IL-6 (R & D Systems Inc., Minneapolis, MN, USA) and for TNF-α, and IL-10 (BD Pharmingen, San Jose, CA, USA) as previously describe^45^. The sensitivity of the assays was 15 pg/mL. The results were normalized by LV total protein^45^.

### Oxidative Stress Profile

Left ventricular tissue was placed in ice-cold buffer and was homogenized in an ultra-Turrax blender using 1 g of tissue for 5 mL of 150 mmol/L potassium chloride and 20 nmol/L phosphate buffer, pH 7.4. Homogenates were centrifuged at 600 g for 10 min at −4 °C. All measurements describe below were performed as previous described^46,47^.

### Antioxidant enzyme activity

The quantification of SOD activity was based on the inhibition of the reaction between O_2_ and pyrogallol. CAT activity was determined by measuring the decrease in H_2_O_2_ absorbance at 240 nm. GPx activity was based on the consumption of NADPH at 340 nm^46,47^.

### Lipid peroxidation by TBARS

For TBARS assay, trichloroacetic acid (10%, w/v) was added to the homogenate to precipitate proteins and to acidify the samples (9). This mixture was then centrifuged (3000 g, 3 min), the protein-free sample was extracted, and *thiobarbituric acid* (0.67%, w/v) was added to the reaction medium. The tubes were placed in a water bath (100 °C) for 15 min. Absorbance was measured at 535 nm using a spectrophotometer^46,47^.

### Protein oxidation

Protein oxidation was measured using a reaction of protein carbonyl groups with 2,4-dinitrofenylhydrazyne to form 2,4-dinitrophenylhydrazone, which can be measured spectrophotometrically. The reaction product was measured at 360 nm^46,47^.

### Statistical analysis

Data analysis was performed using GraphPad Prism (GraphPad Software, La Jolla, California). All data were represented as means ± the standard error of the mean (SEM), the two-way analysis of variance – two-way ANOVA was performed with Turkey’s multiple comparison tests. For nonparametric data, the Kruskal-Wallis test was used. *P* values less than 0.05 were considered significant.
